# Mental health and psychological well-being of Kenyan adolescents from Nairobi and the Coast regions in the context of COVID-19

**DOI:** 10.1186/s13034-023-00613-y

**Published:** 2023-05-19

**Authors:** Gideon Mbithi, Adam Mabrouk, Ahmed Sarki, Rachel Odhiambo, Mary Namuguzi, Judith Tumaini Dzombo, Joseph Atukwatse, Margaret Kabue, Paul Mwangi, Amina Abubakar

**Affiliations:** 1 Institute for Human Development, Aga Khan University, Nairobi, Kenya; 2 School of Nursing and Midwifery, Aga Khan University, Kampala, Uganda; 3 Family and Youth Health Initiative (FAYOHI), Jigawa State, Dutse, Nigeria; 4 Neurosciences Group, KEMRI-Wellcome Trust Research Programme, Centre for Geographic Medicine Research (Coast), Kilifi, Kenya; 5 Department of Psychiatry, University of Oxford, Oxford, UK

## Abstract

**Background:**

Despite the high burden of mental health problems during adolescence and its associated negative consequences, it has remained neglected especially in sub-Saharan Africa. The 2019 novel Coronavirus disease (COVID-19) pandemic has placed additional stress on adolescent mental health. However, there are few studies documenting the burden of mental health problems and even fewer mental health services in the region. In relation to the limited body of knowledge, the present study aims to determine the psychological well-being of adolescents and to assess the risks and associated factors of mental health problems among adolescents in the context of COVID-19 pandemic in Kenya.

**Methods:**

We conducted a cross-sectional survey in 2022 among adolescents aged 13–19 years living in Nairobi, and the Coast region of Kenya. We utilized standardized psychological assessment tools including the Patient Health Questionnaire, Generalized Anxiety Scale, Strengths and Difficulties Questionnaire, The World Health Organization- Five Well-Being Index Scale, and the Pandemic Anxiety Scale, to evaluate the psychological wellbeing of the adolescents. A linear regression model was used to evaluate the correlates associated with quality of life, pandemic anxiety, and emotional and behavioural problems among adolescents. Subsequently, a logistic regression model was used to assess factors associated with depression and general anxiety disorders. Variables with a p-value < 0.25 in the univariate model were included in the multivariable regression model.

**Results:**

The results are based on 797 participants who met the inclusion criteria. We found the prevalence of depression to be relatively higher among out-of-school adolescents at 36.0% compared to school-going adolescents at 20.6%. Furthermore, out-of-school adolescents had significantly higher anxiety scores when compared to their school-going counterparts (27.7% vs 19.1%) respectively. In-school adolescents had a better quality of life scores, lower pandemic anxiety scores, and lower emotional and behavioral problems scores compared to their out-of-school counterparts. Key risk factors associated with depression include; being out-of-school (OR = 1.96 (95% CI 1.33- 2.88) p-value = 0.001), loneliness (OR = 10.68 (95% CI 4.49–22.86) p-value < 0.001), and living in an unsafe neighborhood (OR = 2.24 (95% CI 1.52–3.29) p-value < 0.001). An older age (OR = 1.16 (95% CI 1.03–1.30) p-value = 0.015), being out-of-school (OR = 1.81 (95% CI 1.19–2.77) p-value = 0.006), and living in an unsafe neighborhood (OR = 2.01 (95% CI 1.33–3.04) p-value = 0.001 were key factors associated with anxiety. Furthermore, key factors positively correlated with quality of life include; high socioeconomic status (*ß* (Std.Err) = 0.58 (0.14) p-value < 0.001, talking to friends often (*ß* (Std.Err) = 2.32 (0.53) p-value < 0.001, and being close to parents (*ß* (Std.Err) = 1.37 (0.62) = 0.026.

**Conclusion:**

Our findings imply that mental health support services targeting adolescents in the country should be prioritized, especially for those who are out-of-school.

**Supplementary Information:**

The online version contains supplementary material available at 10.1186/s13034-023-00613-y.

## Background

The burden of mental health problems particularly among adolescents from sub-Saharan Africa (SSA) remains high [15]. Factors including older age, female gender, food insecurity, poor access to health care, and substance abuse have been associated with an increased risk of poor mental health among adolescents in SSA [28]. The rapid physical, social, emotional, behavioural, and cognitive development during adolescence also predisposes the group to a wide range of mental health issues [47]. Additionally, child-parent relationships, including parents’ physical violence toward adolescents also significantly increase the risk of mental health problems among adolescents [26, 28]. The Coronavirus disease 2019 (COVID-19) also placed additional stress on adolescents mental health. Despite the high burden of mental health problems during adolescence and its associated negative consequences, it has remained a neglected issue in SSA. Furthermore, mental health services are scarce in the region [21].

During the onset of the COVID-19 pandemic, the Kenyan government implemented several measures to prevent the spread of the virus. The first case of COVID-19 was reported in Kenya in March 2020, and by April, the government had imposed a lockdown in several regions, including the capital city, Nairobi. Schools were closed, as well as non-essential businesses were shut down. Also, the government imposed a nighttime curfew and limited public gatherings, such as religious events, marriages, and funerals. The impact of these measures on the population was significant, with many people experiencing job loss, reduced income, and difficulty accessing necessities [25]. Schools in the country weren't reopened until January 2021. Millions of students' educations were also impacted by the school closures, raising questions about the long-term implications on their academic development.

School closures and social distancing likely resulted in increased social isolation and loneliness. A systematic review of 83 articles reported that the duration of loneliness during the COVID-19 period was strongly associated with mental health problems [19]. According to the review, social isolation and loneliness increase one's risk of developing depression and anxiety. Positive correlations between social isolation and risky behaviours for eating disorders, self-harm, and suicidal thoughts have also been observed [19]. A study that conducted a meta-analysis to assess the prevalence of depressive and anxiety disorders among children and adolescents globally reported a pooled prevalence of 25.2% and 20.5% for depression and anxiety symptoms respectively [37]. However, none of the 29 studies included in the analysis was from Africa.

Moreover, while Kenya has long struggled with the issue of out-of-school adolescents, the pandemic has exacerbated the problem, resulting in a substantial surge in school dropouts among this demographic in Kenya [50]. Although schools have reopened, many adolescents have not returned due to the financial difficulties faced by their families [34]. The pandemic caused economic hardship for many families in Kenya, leading to some parents being unable to afford school fees or provide the necessary educational resources for their children [34]. School closures, economic hardship, and increased responsibilities at home have contributed to adolescents dropping out of school. This has had significant implications for the mental health and general well-being of these adolescents. School dropouts are at an increased risk of mental health problems, including depression, anxiety, and stress [32]. The long-term consequences of school dropout due to COVID-19 may extend beyond the immediate crisis and have lasting effects on adolescents' mental health and future opportunities. Thus, it is essential to address the plight of the out of school adolescents in Kenya and provide support including mental health care to affected adolescents to mitigate the impact of the pandemic on their well-being.

The present study aims to determine the psychological well-being and the burden of mental health problems among adolescents in Kenya in the context of COVID-19. Specifically, the study aims to assess the quality of life, depressive symptoms, anxiety, and emotional and behavioural problems of school-going adolescents compared to their out-of-school peers. The study also aims to identify risk and protective factors of mental health problems among adolescents. This study's findings provide valuable insights on how disruptions as a result of the COVID-19 pandemic impacted adolescent mental health in Kenya and highlight the urgent need for mental health support and interventions for this vulnerable population.

## Methodology

### Study design and study area

We conducted a cross-sectional study in both Nairobi and the Coast region of Kenya. These regions were selected since they have unique socioeconomic characteristics that are representative of the country’s diversity. The regions have predominantly youthful populations [17]. Nairobi, which is the capital city and the economic hub of Kenya, has a diversified population made up of people who have migrated from different parts of the country in quests of better prospects. On the other hand, the Coast region has both urban and rural populations, with a significant Muslim community. We chose the two regions in order to capture some of the heterogeneity in the country.

The study was conducted between February and May 2022. In Nairobi County, this study primarily focused on Dagoretti South, which is one of the county's seventeen sub-counties. The area is peri-urban and it consists of five wards including; Riruta, Waithaka, Ngando, Mutuini, and Uthiru-Ruthimitu.

In the Coast region, this study was conducted in Kilifi and Mombasa Counties. Mombasa County, is a major transportation and tourism hub in the country. The county has over 250,000 people (43.8%) between the ages of 15 and 35 [6]. In Mombasa County, the study was conducted in Jomvu sub-county. The sub-county is characterized with informal settlements, including Bangladesh which is one of the oldest and largest informal settlements in Mombasa. Kilifi County is situated North of Mombasa. It lies on the shores of the Indian Ocean and is a major tourist destination in the country mostly due to its sandy and beautiful beaches. In Kilifi, the study was conducted in Kaloleni sub-county, which is primarily rural offering a unique perspective on the region's social and economic dynamics.

### Eligibility criteria, and participants’ recruitment

During the recruitment, adolescents and their parents were approached by the study mobilizers in their homes and informed about the study. The mobilizers scheduled appointments for the full consenting and the assessments in advance for the potential participants based on the inclusion criteria.

Adolescents were included in the study if;

1. They were aged 13–19 years as established by their birth certificates.
2. Parents of those aged below 18 years could provide parental consent (or caregiver consent in case the parent is absent) and the adolescents could assent.
3. Those aged at least 18 years had identification cards and could self-consent.
4. If they were currently school-going or had dropped out of schools.
5. Could speak either English or Swahili (the official languages of Kenya).

### Sample size

Sample size calculations were based on the formula for comparison of the prevalence of two samples [7, 36]. We considered a 95% confidence interval (CI), a design effect of 2, a precision of 0.05, and a non-response rate of 1%, leading to a target sample of 724 participants. Taking into account missing data, participants not meeting the inclusion criteria or other factors that can reduce the final sample size we aimed to recruit a sample of > 750. This was also considered sufficient to conduct sub-group analysis and detect prevalence differences between the school-going and out-of-school populations.

**Fig. 1:**
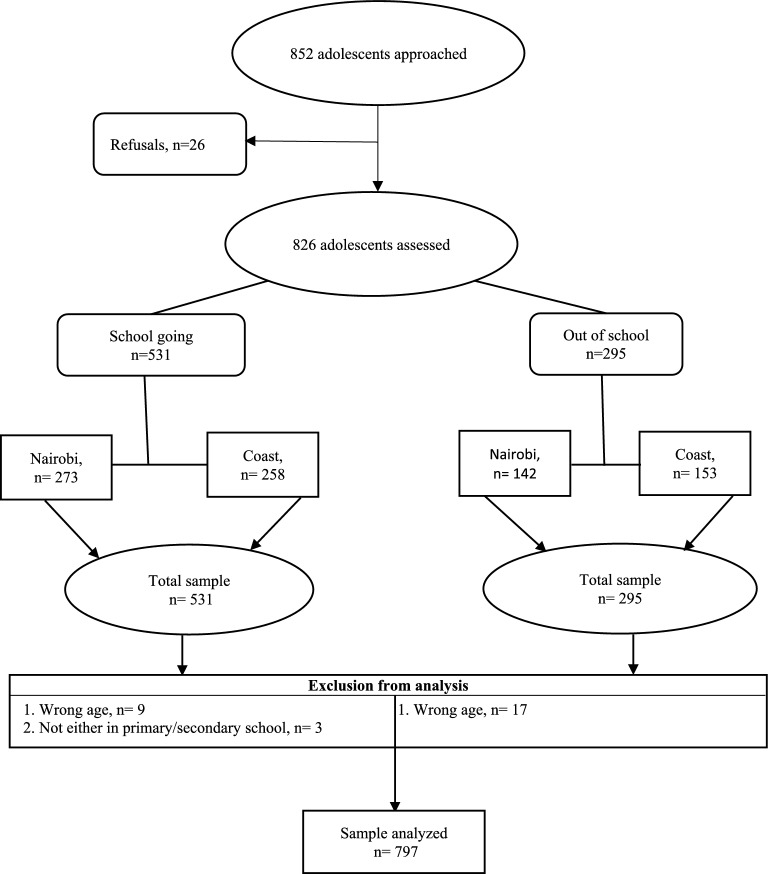
Participants recruitment flowchart

Eight hundred and fifty-two (852) adolescents were approached to participate in the study. Of those, 26 refused to take part in the study. Furthermore, 29 did not meet the full inclusion criteria. Among the participants who completed the baseline survey, 797 were included in the statistical analysis (Fig. 1).

### Data collection procedures

Ten research assistants with either diploma or bachelor’s degree qualifications were recruited and trained before conducting the field activities. The authors' GM, RA, AM, MK, and AA facilitated the week-long training in both sites (Nairobi and Coast). Topics covered during the training sessions included: an overview of constructs, a review of tools/questionnaires, psychological first aid, consent, privacy, confidentially and ethics, how to conduct face-to-face interviews, recruitment and mobilization strategy, COVID-19 protocols, and data management. The team spractised how to effectively conduct consent on participants through role plays. Pretesting of the tools used in the quantitative study was done through a pilot study.

For school-going adolescents, we created a list of schools within our study sites and used simple random sampling to select a total of 10 schools (out of a list of all existing schools including primary, and secondary schools, stratified in such a way that schools are geographically spread out). Since the field activities were conducted when schools were closed for holidays, we worked with a School Health Focal Person (SHFP) from the Ministry of Education (MOE), teachers, community health volunteers (CHVs), and community health assistants (CHAs) to recruit students from the communities who met our inclusion criteria and where from the schools selected.

For the out-of-school adolescents, we worked with the Community Focal Person (CFP) from the county government, CHAs, and CHVs  to recruit participants who met our inclusion criteria. Out-of-school adolescents were defined as adolescents who were not enrolled in either primary or secondary schools [14]. Adolescents who had completed primary school or secondary school exams in February and March 2022 respectively and were waiting for the government directive to advance to the next level of their education were not considered to be part of the out-of-school population. However, those who had completed primary-level national exams before 2022 and had not progressed to secondary or high school at the time of data collection were deemed out of school. Before the mobilization, all the mobilizers were trained on the study’s mobilization and community engagement strategy and procedures. We drew on the CHVs' and CHAs' knowledge of both the geography and local population of the study sites to identify potential out-of-school adolescents.

During our face-to-face data collection, we ensured all the stipulated COVID-19 guidelines were followed and data collection was carried out in specified sites in each of the counties. Adolescents below eighteen years were accompanied by their parents to ensure they provide consent. The assessments took approximately one hour. After the assessment, a small refreshment was provided to the participants. To ensure that the refreshments offered did not influence participation or responses, we made it clear to all of the participants that the refreshments were being offered as a token of appreciation for their time and effort and that their decision to participate in the study would not be influenced by whether or not they accepted the refreshments. Additionally, refreshments were offered to all mobilized participants, regardless of whether or not they agreed to participate in the study. This ensured that there was no differential treatment of participants based on their willingness to participate. Finally, we used standardized questionnaires and a data collection process, which was administered by trained research assistants who were not involved in the distribution of the refreshments. This minimized the potential for response bias or influence on the data collection process.

Cost incurred such as travel to attend these discussions were reimbursed to the guardians/parents based on standard acceptable rates. Based on various sources of information, we determined a flat rate of transportation rate. This included conducting planning engagements in which we consulted with community leaders to obtain an estimate of the typical cost of transportation in the study area. Secondly, we reviewed previous studies conducted in the area that involved similar participant populations and assessed the transportation costs associated with those studies. The rate agreed upon was also per rates as guided by the Aga Khan University Institute for Human Development participant’s transport payment and reimbursement standard operating procedures. We aimed to ensure that the transportation reimbursement provided was sufficient to cover the cost of transportation to and from the study site, without providing an excessive or undue financial incentive for participation.

### Measures

#### Social demographic measure

The information on adolescents' age, gender, school attendance (whether they were in school or not), level of education, religion, economic status, and parental marital status was collected using a socio-demographic survey tool. The household socioeconomic status was evaluated by asking the adolescents about items found in their homes using an assets index that has been used in similar studies focusing on adolescents [1, 39].

#### Patient health questionnaire- 9 (PHQ-9)

PHQ-9 is a multipurpose instrument for screening, diagnosing, and measuring the severity of depression [18]. In this study, PHQ-9 was used to screen for depressive symptoms. The tool consists of 9 items scored on a 4-point Likert scale (0 = not at all, 1 = several days, 2 = more than a week, 3 = nearly every day). The items are summated together, and the total scores range from 0–27. A score of 5–9, 10–14, 15–19, and 20–27 represents the cut-off points for mild, moderate, moderately severe, and severe depression, respectively. PHQ-9 has been used and validated for use in adolescents from Africa [2, 20, 43]. The tool has been validated in the Swahili language [24]. Psychometric analysis performed on the PHQ-9 scale for the current study showed a relatively high internal reliability of the scale (Cronbach’s alpha = 0.83).

#### General anxiety disorder- 7 (GAD-7)

The GAD-7 tool was used to assess anxiety among adolescents. The tool consists of seven items, with responses on a Likert scale ranging from 0 (not at all) to 3 (more than half the days) [38]. The scale's total score ranges from 0 to 21, with cutoffs of 5–9, 10–14, and 15–21 equating to mild, moderate, and severe anxiety symptoms, respectively. GAD-7 has been extensively used [, 3, 30] and has been validated in similar settings while maintaining its unidimensional latent structure with favourable psychometric characteristics [27]. In this study, results from reliability analysis showed that the GAD-7 scale had good internal reliability (Cronbach’s alpha = 0.81).

#### Strengths and difficulties questionnaire (SDQ)

The SDQ is a brief 25-item emotional and behavioural screening tool for children and young people [12]. The SDQ questionnaire contains 25 items, grouped into five subscales (emotional symptoms, conduct issues, hyperactivity/inattention, peer relationship issues, and prosocial behaviour) and the item scores are 0 (not true), 1 (somewhat true), and 2 (certainly true). The scores of all except the prosocial behaviour sub-scale (positive measure) are aggregated to give a total difficulty score ranging from 0 to 40. SDQ has been widely used in Africa to assess internalizing and externalizing problems among children and adolescents, in which as of 2018 it had been used in 54 studies conducted across 12 countries in Africa, including Kenya [13]. A reliability analysis was carried out including 20 items from the SDQ scale. The analysis showed that the scale had acceptable internal reliability (Cronbach’s alpha = 0.75).

#### Pandemic anxiety scale (PAS)

The PAS was used to identify the specific aspects of the COVID-19 pandemic that cause worries. The PAS is a nine-item scale with ratings on a five-point Likert scale of 0 (strongly disagree) to 4 (strongly agree), with scores ranging from 0 to 36. The scale includes questions about the disease itself as well as concerns and worries about the pandemic's consequences and has good validity and reliability [22]. Item 7 *(I am worried about missing schoolwork)* was not used in the analysis as it was not relevant to the out-of-school population, hence the highest score based on the remaining eight questions was 32. The scale had acceptable internal reliability based on results from psychometric analysis (Cronbach’s alpha = 0.77).

#### The World Health Organization- five well-being index (WHO-5)

The WHO-5 scale was administered to assess the adolescents' quality of life. The tool has five items scored from 0 (at no time) to 5 (all the time), and the total score ranges from 0 to 25 [46]. A total score of 0 illustrates the worst possible quality of life, while a total score of 25 represents the best probable quality of life. The tool has been used in a similar setting, validated in the Swahili language [5], and has shown good construct validity in measuring the quality of life in younger populations [44]. Psychometric analysis of the current study showed that the WHO-5 tool had acceptable internal reliability (Cronbach’s alpha = 0.75).

#### Covid-19 related questionnaire

We administered the questionnaire to evaluate how the COVID-19 pandemic had affected adolescent mental health care-seeking behaviour, school attendance, peer relationships in the school setting, and interactions with parents.

#### Assembled items for the Kilifi adolescent health risk behavior questionnaire

The tool was used to assess health risk behaviours among adolescents such as alcohol and drug abuse use, sexual behaviours, and violence among others. The tool is easy to use and has acceptable test–retest reliability (Gwet’s AC1 = 0.82) [40].

### Data management

All data were collected using Open Data Kit (ODK) through tablets and computers. These electronic instruments were password protected and encrypted to avoid data loss and were only available to authorized personnel. To ensure quality control and safety, the data manager double-checked any inconsistencies in the data before uploading it to the server daily.

### Statistical analysis

Stata version 17, a statistical software, was used to analyze all the data [41]. The sample characteristics of the adolescents were summarized using proportions, means, and standard deviations. The differences between the in-school and out-of-school groups were investigated using the independent t-test for continuous variables and Pearson's chi-squared test for categorical variables. For depression and anxiety, the point prevalence and corresponding 95% confidence intervals (95% CI) were calculated. The figures and tables were used to present the results.

A linear regression model was used to evaluate the socio-demographic characteristics, COVID-19-related questions, parents and peer relationships, psychosocial stressors associated with quality of life, pandemic anxiety, and emotional and behavioural problems. First, a univariate linear model was fitted for each outcome, and the variables with a *p*-value < 0.25 were included in the multivariable linear regression model [4]. Plots and statistical tests were used to evaluate the model assumptions; linearity, normality, multicollinearity, homoscedasticity, and the presence of outlying observations [11].

Subsequently, a logistic regression model was used to assess factors associated with depression and general anxiety disorders. A univariate logistic model was fitted, and variables with a *p*-value < 0.25 were included in the multivariable logistic regression model. The odds ratios (ORs) with corresponding 95% confidence intervals (CIs) and associated *P-*values were reported. A 5% significance level was used for this study. Furthermore, in the present study, Cronbach’s coefficient α was used to calculate the internal reliability coefficients of the items used in the regression models. A score of > 0.70 was considered an acceptable value of Cronbach’s alpha based on the reliability analysis [42].

## Results

A total of 852 participants were approached to participate in the study, however, our analysis was based on 797 who met the full inclusion criteria (see Fig. 1). Table 1 shows the socio-demographic characteristics of the study participants. Additionally, statistical group comparisons between school-going and out-of-school adolescents are presented. The participants in the study had an average age of 16.3 years (*SD* = 1.9). Out-of-school adolescents had a higher mean age (*M* = 16.6, *SD* = 2.0) than in-school adolescents (*M* = 16.1, *SD* = 1.8), *p* < 0.001. There is no significant difference in terms of the number of participants in the two geographical regions [*P* = 0.190]. The majority of the participants were Christians (75.2%), while 59.5% were female, 53.6% were in secondary schools, and 50.1% were from the coastal region. Furthermore, 4.6% of adolescents were parents, and 1.7% of females were pregnant, with most of them from the out-of-school group. Adolescents who attended school had a higher mean social-economic status score (*M* = 3.2, *SD* = 1.6) compared to those who did not attend (*M* = 2.8, SD = 1.5), *p* < 0.001. Furthermore, the results show a significant difference in education level, having a child(ren), and being expectant between school-going and out-of-school adolescents.

**Table 1:** Socio-demographic characteristics of participants

	Overall *n (%)*	In school *n (%)*	Out-of-school *n (%)*	*P-*value
Total	797	519 (65.1%)	278 (34.9%)	
Sex				
Female	474 (59.5%)	323 (62.2%)	151 (54.3%)	**0.030**^+^
Male	323 (40.5%)	196 (37.8%)	127 (45.7%)	
Region				
Coast	399 (50.1%)	251 (48.4%)	148 (53.2%)	0.190^+^
Nairobi	398 (49.9%)	268 (51.6%)	130 (46.8%)	
Age *mean* ± *SD*	16.3 ± *1.9*	16.1 ± *1.8*	16.6 ± *2.0*	< 0.001^*^
Level of education				
None	7 (0.9%)	0	7 (2.5%)	**< 0.001**^+^
Primary	363 (45.5%)	192 (37.0%)	171 (61.5%)	
Secondary	427 (53.6%)	327 (63.0%)	100 (36.0%)	
Religion				
Christian	599 (75.2%)	398 (76.7%)	201 (72.3%)	0.238^+^
Islam	193 (24.2%)	119 (22.9%)	74 (26.6%)	
Others e.g. traditional	5 (0.6%)	2 (0.4%)	3 (1.1%)	
Social Economic Status *mean* ± *SD*	3.1 ± *1.6*	3.2 ± *1.6*	2.8 ± *1.5*	< 0.001*
Have any child(ren)				
Yes	37 (4.6%)	7 (1.4%)	30 (10.8%)	**< 0.001**^+^
No	760 (95.4%)	512 (98.6%)	248 (89.2%)	
Currently expectant^a^				
Yes	8 (1.7%)	1 (0.3%)	7 (4.7%)	**< 0.001**^+^
No	462 (97.9%)	321 (99.7%)	141 (94.0%)	
Don’t know	2 (0.4%)	0	2 (1.3%)	

^*a*^*;respondents are only girls;* ± *: Standard deviation;* + ; P-values for binary or categorical variables are from Pearson's chi-squared test *; P-values for the continuous variable are from the student’s t-test

Bold, statistically significant results (*p*-values).

Table 2 summarizes the prevalence estimates for depressive and anxiety symptoms. In terms of severity for both depression and anxiety, the prevalence of the two disorders was relatively higher among out-of-school as compared to in-school adolescents. Using a cut-off score ≥ 10 for PHQ-9, the prevalence of depression among school-going adolescents was 20.6%; relatively lower than the out-of-school at 36.0% [*p* < 0.001]. Similarly, out-of-school adolescents had a significantly higher prevalence of anxiety (27.7%) than in-school adolescents (19.1%), *p* < 0.001. The overall prevalence of positive screen for comorbidity for both depression and anxiety symptoms was 12.5%, with a high prevalence score in out-of-school compared to school-going adolescents (18.3% vs 9.1%, *p* < 0.001).

**Table 2:** Prevalence of common mental disorders among school-going versus out-of-school adolescents

	Overall, *n*= 797		School going adolescents, *n* = 519		Out-of-school adolescents, *n* = 278		*P*- value
	Frequency	Prevalence (95% CI)	Frequency	Prevalence (95% CI)	Frequency	Prevalence (95% CI)	
The severity of depressive symptoms							
None (0–4)	370	46.4 (43.0–49.9)	274	52.8 (48.5–57.1)	96	34.5 (29.2–40.3)	**< 0.001**
Mild (5–9)	220	27.6 (24.6–30.8)	138	26.6 (23.0–30.6)	82	29.5 (24.4–35.1)	
Moderate (10–14)	132	16.6 (14.1–19.3)	69	13.3 (10.6–16.5)	63	22.7 (18.1–28.0)	
Moderately severe (15–19)	56	7.0 (5.4–9.0)	28	5.4 (3.7–7.7)	28	10.1 (7.0–14.2)	
Severe (20–27)	19	2.4 (1.5–3.7)	10	1.9 (1.0–3.5)	9	3.2 (1.7–6.1)	
Positive depression screen (cut-off score ≥ 10)							
Yes	207	26.0 (23.0–29.1)	107	20.6 (17.3–24.3)	100	36.0 (30.5–41.8)	**< 0.001**
The severity of anxiety symptoms							
None (0–4)	472	59.2 (55.8–62.6)	322	62.0 (57.8–66.1)	150	54.0 (48.0–59.8)	**< 0.001**
Mild (5–9)	173	21.7 (19.0–24.7)	122	23.5 (20.0–27.4)	51	18.3 (14.2–23.4)	
Moderate (10–14)	117	14.7 (12.4–17.3)	57	11.0 (8.6–14.0)	60	21.6 (17.1–26.8)	
Severe (15–21)	35	4.4 (3.2–6.1)	18	3.5 (2.2–5.4)	17	6.1 (3.8–9.6)	
Positive anxiety screen (cut-off score ≥ 10)							
Yes	152	19.1 (16.5–22.0)	75	14.5 (11.7–17.8)	77	27.7 (22.7–33.3)	**< 0.001**
Positive screen for comorbid depressive, and anxiety symptoms							
Yes	100	12.5 (10.4–15.0)	49	9.1 (8.8–9.3)	51	18.3 (14.2–23.3)	**< 0.001**

P-values for the binary/categorical variables are from Pearson's chi-squared test. Bold, statistically significant results (*p*-values)

**Fig. 2:**
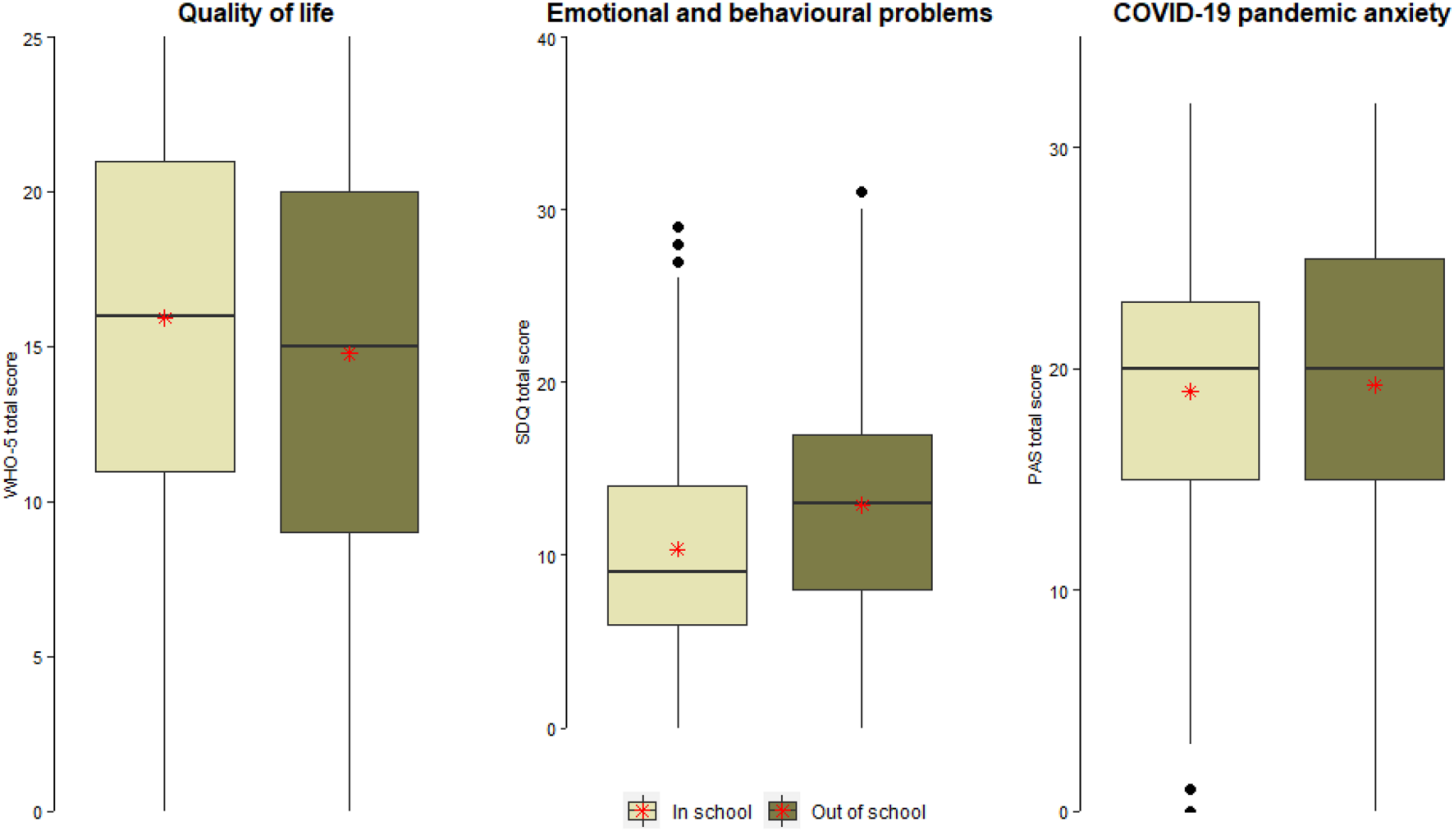
Emotional and behavioural problems, pandemic anxiety, and quality of life of school going versus the out-of-school adolescents

Figure 2 represents the box plots of emotional and behavioural problems, pandemic anxiety, and quality of life among school-going, and out-of-school adolescents. The red asterisk represents the mean score, while the solid black line between the boxes represents the median score. The in-school adolescents had significantly higher quality of life scores (*M* = 15.9, *SD* = 6.1) compared to their out-of-school counterparts (*M* = 14.8, SD = 6.6), *t*(795) = 2.49, *p* = 0.01. The effect size (Cohen's d) was small, with a value of 0.18, 95% CI [0.03, 0.32].

Furthermore, for emotional and behavioural problems, the mean score for out-of-school adolescents was (*M* = 12.9, *SD* = 6.3), while for the in-school adolescents was (*M* = 10.4, *SD* = 5.9), *t*(795) = − 5.68, p < 0.001, Cohen’s d = 0.41, 95% CI [0.27, 0.56]. This indicates a moderate effect size, with out-of-school adolescents having statistically significantly higher levels of emotional and behavioural problems than in-school adolescents.

There was no significant difference in terms of pandemic anxiety mean scores between the out-of-school (*M* = 19.3, *SD* = 6.8) and in-school groups (*M* = 19.0, *SD* = 6.1), *t*(795) = − 0.57, *p* = 0.572, Cohen's *d* = 0.05, 95% CI [− 0.09, 0.19]. This indicates a very small effect size, suggesting that the pandemic anxiety mean scores for the two groups were similar.

Additional file 1: Table S1 illustrates results from univariate logistic regression model analyses assessing the correlates i.e., both risk and protective factors associated with depression and anxiety. Two socio-demographic characteristics including being out of school and an increase in age were associated with higher odds of both depression and anxiety (Additional file 1: Table S1). An adolescent having a child was significantly associated with higher odds of anxiety but not depression. For COVID-19-related questions, having a COVID-19 infection, and having someone who is close to you including a family member or a friend being infected with COVID-19 were factors significantly associated with higher odds of depression and anxiety (Additional file 1: Table S1). For parents and peer relationship questions, interacting less with friends or peers, loneliness, and arguing with parents often were all significantly associated with higher odds of depression and anxiety (Additional file 1: Table S1. Furthermore, living in unsafe neighbourhoods, being physically forced to have sex, and drinking alcohol were also significantly associated with higher odds of depression and anxiety. Being Muslim, being very close to parents, and talking to friends often, were factors significantly associated with lower odds of both anxiety and depression (Additional file 1: Table S1).

Additional file 2: Table S2 presents the results of univariate linear model analyses showing the association of factors (both predictors and protective factors) and quality of life, pandemic anxiety, and emotional and behavioural problems as outcomes. Factors positively correlated with quality of life include; being Muslim, having high socioeconomic status, talking to friendsoften, and having a close relationship with parents (Additional file 2: Table S2). Factors negatively correlated with quality of life include; an increase in age, being out-of-school, adolescents having children, interacting less with friends, loneliness, arguing with parents often, feeling unsafe, physically forced to have sex, and drinking alcohol (Additional file 2: Table S2.

Talking to friends often was the only factor that was negatively correlated with both pandemic anxiety and emotional and behavioural problems (Additional file 2: Table S2). Being close to parents, and being Muslim were factors negatively correlated with emotional and behavioural problems. Factors associated positively with both pandemic anxiety and emotional and behavioural problems include being female, increase in age, having a someone close being infected with COVID-19, interacting less with peers, loneliness, feeling unsafe, and physically being forced to have sex (Additional file 2: Table S2). Furthermore, an adolescent having children was a predictor of pandemic anxiety but not emotional and behavioural problems (Additional file 2: Table S2). Being out of school, arguing with parents, and history of drinking alcohol were positively correlated with emotional and behavioural problems but not pandemic anxiety (Additional file 2: Table S2).

Table 3 shows the results of the multivariate regression model showing both protective and predictor factors associated with depression, anxiety, emotional and behavioural problems, quality of life, and pandemic anxiety. Predictors associated with high odds of depression include; being out of school loneliness, and living in an unsafe neighbourhood (Table 3). Talking to friends often, being Muslim, and being close to parents were factors associated with lower odds of depression (Table 3). Additionally, an increase in age, being out of school, interacting less with friends, loneliness, and living in an unsafe neighbourhood were factors associated with higher odds of anxiety (Table 3). Factors negatively correlated with quality of life include; an increase in age, loneliness interacting less with friends, and living in an unsafe neighbourhood (Table 3). Furthermore, factors positively correlated with quality of life include; being Muslim, having high socioeconomic status, talking to friends often, and being close to one’s parents (Table 3). Loneliness was the only factor positively correlated with pandemic anxiety (Table 3). We didn’t identify any factor that was negatively correlated with pandemic anxiety (Table 3). Being out of school, loneliness arguing with parents often, and living in an unsafe neighbourhood were factors positively correlated with emotional and behavioural problems (Table 3). Belonging to other religions e.g. traditional religions was the only factor found to be negatively correlated with emotional and behavioural problems (Table 3).

**Table 3:** Results from regression analysis showing factors associated with depression, anxiety, pandemic anxiety, emotional behavioural problems and quality of life

	Depression		Anxiety		Pandemic anxiety		Emotional and behavioural problems		Quality of life	
	*(OR:95% CI)*	*P*-value	*(OR:95% CI)*	*P*-value	*ß* (Std.Err)	*P*-value	*ß* (Std.Err)	*P*-value	*ß* (Std.Err)	*P*-value
Socio-demographic										
Sex										
Male	*Reference*									
Female	–	–	–	–	− 0.77 (0.46)	0.095	− 0.63 (0.37)	0.088	–	–
Age	1.08 (0.97–1.20)	0.149	1.16 (1.03–1.30)	**0.015**	0.21 (0.12)	0.094	0.13 (0.10)	0.196	− 0.32 (0.14)	**0.023**
Schooling										
Yes	*Reference*									
No	1.96 (1.33–2.88)	**0.001**	1.81 (1.19–2.77)	**0.006**	–	–	1.58 (0.38)	**< 0.001**	− 0.52 (0.48)	0.284
Level of education										
None	*Reference*									
Primary	–	–	–	–	–	–	–	–	− 2.46 (2.15)	0.253
Secondary	–	–	–	–	–	–	–	–	− 2.90 (2.19)	0.186
Religion										
Christian	*Reference*									
Islam	0.62 (0.38–1.00)	**0.049**	0.72 (0.42–1.23)	0.230	− 0.02 (0.53)	0.975	− 0.19 (0.43)	0.658	1.76 (0.50)	**< 0.001**
Others e.g. traditional	0.37 (0.04–3.33)	0.377	0.73 (0.07–7.46)	0.793	− 2.13 (2.85)	0.456	− 6.22 (2.26)	**0.006**	4.42 (2.57)	0.085
Social Economic Status	–	–	0.90 (0.78–1.03)	0.130	–	–	− 0.15 (0.12)	0.231	0.58 (0.14)	**< 0.001**
Have any child(ren)										
No	*Reference*									
Yes	0.88 (0.38–2.03)	0.773	1.19 (0.51–2.74)	0.691	1.71 (1.08)	0.114	–	–	− 0.80 (0.99)	0.416
COVID-19 related questions										
Receive support before lockdown (Mental health support, support from social services, educational support)										
Yes	*Reference*									
No	–	–	–	–	–	–	–	–	0.60 (0.43)	0.166
Having COVID-19 infection										
No	*Reference*									
Yes	3.52 (0.81–15.38)	0.094	2.57 (0.62–10.60)	0.192	–	–	2.42 (1.47)	0.101	-1.74 (1.66)	0.297
Having someone close infected with COVID-19 e.g. A friend, a family member										
No	*Reference*									
Yes	1.42 (0.86–2.33)	0.166	1.38 (0.82–2.35)	0.229	0.60 (0.63)	0.341	0.63 (0.52)	0.220	− 0.19 (0.58)	0.744
Parents and peer relationships										
Hear from or talk to your friends										
Rarely	*Reference*									
Occasionally	0.55 (0.35–0.85)	0.007	0.69 (0.43–1.12)	0.133	0.14 (0.55)	0.804	− 0.75 (0.44)	0.087	0.24 (0.49)	0.628
Frequently	0.60 (0.37–0.96)	**0.033**	0.74 (0.44–1.24)	0.248	− 0.71 (0.59)	0.231	− 0.65 (0.47)	0.167	2.32 (0.53)	**< 0.001**
Change of interaction with peers since last week										
No- it is the same	*Reference*									
Yes- I interact with them less	1.40 (0.92–2.12)	0.116	1.56 (1.00–2.43)	**0.049**	0.29 (0.53)	0.591	0.74 (0.43)	0.081	− 1.08 (0.48)	**0.024**
Yes- I interact with them more	0.83 (0.48–1.44)	0.506	0.90 (0.49–1.65)	0.729	0.57 (0.65)	0.382	0.20 (0.52)	0.704	0.74 (0.59)	0.206
Feeling lonely, lacking company, feeling left out or isolated										
Not at all	*Reference*									
Sometimes	2.14 (1.43–3.19)	**< 0.001**	2.08 (1.35–3.23)	**0.001**	1.93 (0.52)	**< 0.001**	3.58 (0.42)	**< 0.001**	− 2.99 (0.47)	**< 0.001**
Always	10.68 (4.99–22.86)	**< 0.001**	5.96 (2.99–11.87)	**< 0.001**	3.87 (0.96)	**< 0.001**	6.74 (0.77)	**< 0.001**	− 2.65 (0.87)	**0.002**
Close with your parents										
Not very close	*Reference*									
Fairly close	0.36 (0.19–0.68)	**0.002**	0.74 (0.38–1.44)	0.376	0.41 (0.83)	0.622	− 1.84 (0.66)	**0.006**	0.57 (0.75)	0.444
Very close	0.39 (0.24–0.64)	**< 0.001**	0.62 (0.36–1.06)	0.079	− 0.27 (0.68)	0.691	− 2.93 (0.54)	**< 0.001**	1.37 (0.62)	**0.026**
Argue with parent										
Never	*Reference*									
Occasionally	0.77 (0.48–1.23)	0.278	0.84 (0.51–1.38)	0.486	–	–	0.44 (0.45)	0.321	− 0.76 (0.50)	0.133
Frequently	1.28 (0.78–2.10)	0.327	1.26 (0.75–2.13)	0.380	–	–	1.80 (0.52)	**0.001**	− 0.89 (0.58)	0.127
Psychosocial stressors questions										
Feeling unsafe No	*Reference*									
Yes	2.24 (1.52–3.29)	**< 0.001**	2.01 (1.33–3.04)	**0.001**	0.84 (0.50)	0.096	3.00 (0.41)	**< 0.001**	**− 1.45 (0.46)**	**0.002**
Physically forced to have sex										
No	*Reference*									
Yes	1.14 (0.57–2.27)	0.706	1.27 (0.64–2.51)	0.491	0.09 (0.91)	0.921	0.46 (0.73)	0.530	0.28 (0.82)	0.733
Drunk alcohol (at least a bottle within a month ago)										
No	*Reference*									
Yes	1.18 (0.66–2.13)	0.572	1.20 (0.65–2.22)	0.550	–	–	0.82 (0.61)	0.174	− 0.86 (0.68)	0.206

Bold, statistically significant results (*p*-values)

## Discussion

The current study provides timely insights into the psychological well-being and the burden of mental health problems among adolescents in Kenya in the context of COVID-19. Across the sample, the overall prevalence of depression and anxiety was 26.0% and 19.1% respectively. These findings are consistent with other studies conducted in the context of COVID-19. A recent systematic review of studies from sub-Saharan Africa of adolescents including out-of-school adolescents and those from a poor background reported a pooled prevalence of depression and anxiety at 29.0% and 19.3% respectively [15]. Additionally, one in every four youths globally is thought to experience elevated depression symptoms, while one in five youths experienced clinically elevated anxiety during the COVID-19 pandemic [37]. When compared to pre-pandemic estimates [8], the burden of mental health problems reported in the current study seems to be relatively higher.

We observe that out-of-school adolescents have relatively poorer outcomes in terms of depression, anxiety, quality of life, and emotional and behavioural problems when compared to their in-school counterparts. Additionally, pandemic anxiety scores are almost equivalent between the two groups suggesting that the two groups experienced comparable levels of worry about the pandemic. Comparative studies are scarce since there is a paucity of research comparing the mental health outcomes of adolescents who attend school and those who do not. Adolescents who are out-of-school face a lot of challenges including struggling more to meet basic needs which makes them more prone to poor mental health. Additionally, the experience of stressful life events, absence of social support, an HIV-positive diagnosis, being a young mother, and teenage pregnancy pregnancies have been associated with higher depressive symptoms among out-of-school adolescents [31]. Schools have a responsibility to play in promoting positive mental health and the general well-being of students. Support from teachers, co-curriculum activities, and support from peers are key components in school that help students deal with difficulties that can lead to mental health problems. Adolescents who are not enrolled in school generally don’t have access to this support even though they face more challenges, which may explain why they have a disproportionately greater burden of mental health problems than their school-going peers.

Our study revealed that age was a major social demographic characteristic associated with high odds of anxiety and poor quality of life. Prior research shows that the symptoms of mental health problems including anxiety may increase with age. Various explanations for this association have been studied. One of the first studies that assessed prevalence rates among adolescents during the early stages of the pandemic, reported that attending senior grades was a risk factor for anxiety and depression compared to attending junior grades [49]. The increased academic pressure that school-going adolescents face when they approach the end of their secondary education exams leads to increased psychosocial stress that exacerbates the symptoms of depression and anxiety [29]. Also, as the age of adolescents increases, they get exposed to greater stresses and factors that might lead to poor mental health, such as peer pressure, increased independence, social adjustment, and familial isolation [23].

The COVID-19 pandemic and its associated measures appear to have negatively impacted adolescents' psychological well-being. The loss of peer interaction including feeling isolated from other was a factor that was significantly associated with both depression and anxiety. Adolescents seem to be more affected by loneliness, as an unintended effect of the pandemic containment measures. This shows that interaction and support from peers are essential for their mental health. Being confined to one's house can disrupt sleep patterns and exercise habits, whereas excessive use of technology can influence a person’s mental health [48]. Furthermore, losing connections with others and feeling alienated and alone, can result in feeling distressed and depressed. Our findings are consistent with previous research on the contribution of COVID-19 to the escalation of mental health problems among adolescents. According to a recent COVID-19 survey on adolescents in Kenya, extended school closures and economic hardships caused many adolescent girls to have teenage pregnancies and early marriages (Population [35]. The study has shown that girls and boys reported increased tension in their households resulting in cases of emotional, physical, and sexual violence (Population [35]. This violence subjected to adolescents as a result of the pandemic had a huge toll on their mental health.

Furthermore, we identified health risk behaviours and psychological stressors associated with mental health outcomes. In our study, girls who reported having experienced sexual violence had higher levels of depression and anxiety as compared to girls who had not reported any experience of sexual violence. This association is in line with existing literature [9]. We found that adolescents who take alcohol had higher levels of depression and anxiety compared to those who had taken none. Studies have reported that during adolescence, several drinking behaviours including drinking alcohol often increase the risk of depressive symptoms among adolescents [33]. The pandemic has also predisposed adolescents to risky behaviours such as drug use including alcohol. A recent study assessing substance use before and during the pandemic among adolescents in Uganda, reported a slight increase in alcohol use although it was not a statistically significant change compared to before the pandemic [16].

We found that adolescents who were extremely close to their parents were more likely to report lower levels of depressive symptoms. Furthermore, the same adolescents also had a better quality of life. This shows the importance of a healthy connection between adolescents, their parents and their families. Our results show that adolescents who belonged to the Islam religion reported lower odds of depression and also a better quality of life. Although this was an interesting finding, we were unable to ascertain the reason behind this observation. However, prior research has demonstrated that religion and spirituality can play a role in promoting positive mental health outcomes, for instance through positive religious coping, social support, and positive attitudes and beliefs [45]. Additionally, adolescents who reported being close and engaging with their friends reported lower odds of depressive symptoms as well. Adolescents who feel close and connected to the people around them are buffered from risk factors around them associated with poor mental health [10]. This shows the importance of promoting positive youth connectedness including connectedness to family and parents, schools, community, and peers. While connections inside and outside the family setting have been linked with positive well-being, the absence of these connections imposes the risk of negative outcomes. This points to the fact that promoting positive connectedness not only reduces potential risks but also promotes positive outcomes among adolescents.

### Strengths and limitations

This study has strengths and limitations worth highlighting. First, it not only focused on school-going adolescents but also adolescents out of school, a population that tends to be ignored yet is at a very high risk of poor mental health. In addition to conducting mental health assessments in the current study, we also offered psychological counselling to adolescents who were experiencing significant psychological distress. Another strength of the study is its ability to explore a timely research theme on assessing the psychological well-being and burden of mental health problems among adolescents in the context of COVID-19. Furthermore, this study used measures that have been psychometrically tested in our setting and have shown to have good internal reliability and validity. Additionally, all assessments were done in a one-on-one environment within private and secure rooms.

In terms of the limitations of the study, data on the outcomes and the correlates were collected based on self-report, which is prone to information and the potential of recall bias. The cross-sectional nature of the study means we cannot establish the causal relationship between the factors assessed and the mental health outcomes. While our study aimed to capture a representative sample of adolescents from two regions in Kenya, we acknowledge that our findings may not be generalizable to the entire Kenyan population. Even with all effort put to minimize bias in the study, we recognize that the refreshments provided to the participants could have influenced their engagement and motivation. We did not collect data on the treatment-seeking behaviours of adolescents with mental health issues, meaning we cannot provide a comprehensive overview of the situation in the country.

## Conclusion

Our analysis revealed a relatively high prevalence of depression, emotional and behavioural problems, and anxiety among out-of-school adolescents when compared to school-going adolescents. There was no significant difference in terms of pandemic anxiety levels in the two groups. School-going adolescents had a better quality of life than the out of school adolescents. We also identified correlates associated with mental health outcomes. These factors include; socio-demographic factors, parents and peer relationships, COVID-19-related factors, and psychosocial stressors and health risk behaviours. This indicates that the predictors of mental health problems among adolescents are multidimensional and interrelated. Given the high burden reported in this study, there is a need to embed mental health services and interventions in primary health care. In the school systems, there is a need to integrate mental health education within the school curriculum.

## Supplementary Information

**Additional file 1: Table S1.** Results from univariate logistic regression showing factors associated with common mental disorders.

**Additional file 2: Table S2.** Results from univariate linear model showing the relationship between predictor variables and pandemic anxiety, quality of life, and emotional and behavioural problems.

## Data Availability

The de-identified dataset used and analysed during this current study will be made available upon reasonable request, in due consideration of Aga Khan University’s data sharing policies. Requests to access the datasets should be directed to amina.abubakar@aku.edu.
