# Supplementary material for: Mental health and psychological well-being of Kenyan adolescents from Nairobi and the Coast regions in the context of COVID-19
Source: Child Adolesc Psychiatry Ment Health. 2023 May 19;17:63. doi: 10.1186/s13034-023-00613-y (PMC10198601; doi:10.1186/s13034-023-00613-y)
Supplement: Supplementary file 1 — Additional file 1: Table S1. Results from univariate logistic regression showing factors associated with common mental disorders. [file 13034_2023_613_MOESM1_ESM.docx]

**Additional table 1:** Results from univariate logistic regression showing factors associated with common mental disorders

|  | **Depression** | | | **Anxiety** | | |
| --- | --- | --- | --- | --- | --- | --- |
|  | ***(OR:95%CI)*** | ***P*-value** | | | ***(OR:95%CI)*** | ***P*-value** |
| **Socio-demographic** |  | |  |  |  |  |
| **Sex** |  | |  |  |  |  |
| Male | *Reference* | |  |  |  |  |
| Female | 1.11 (0.80-1.54) | | 0.522 |  | 1.06 (0.74-1.51) | 0.769 |
| **Age** | 1.13 (1.04-1.23) | | **0.006** |  | 1.20 (1.09-1.33) | **<0.001** |
| **Schooling** |  | |  |  |  |  |
| Yes | *Reference* | |  |  |  |  |
| No | 2.16 (1.56-2.99) | | **<0.001** |  | 2.27 (1.58-3.25) | **<0.001** |
| **Level of education** |  | |  |  |  |  |
| None | *Reference* | |  |  |  |  |
| Primary | 2.13 (0.25-17.90) | | 0.487 |  | 1.38 (0.16-11.68) | 0.300 |
| Secondary | 2.11 (0.25-17.70) | | 0.492 |  | 1.45 (0.17-12.19) | 0.340 |
| **Religion** |  | |  |  |  |  |
| Christian | *Reference* | |  |  |  |  |
| Islam | 0.56 (0.37-0.84) | | **0.005** |  | 0.63 (0.40-0.99) | **0.045** |
| Others e.g. traditional | 1.68 (0.28-10.16) | | 0.571 |  | 2.58 (0.43-15.61) | 0.302 |
| **Social Economic Status** | 0.95 (0.86-1.05) | | 0.303 |  | 0.93 (0.83-1.05) | 0.232 |
| **Have any child(ren)** |  | |  |  |  |  |
| No | *Reference* | |  |  |  |  |
| Yes | 1.58 (0.79-3.16) | | 0.196 |  | 2.13 (1.04-4.33) | **0.038** |
| **COVID-19 related questions**  **Receive support before lockdown (Mental health, support from social services, educational support)** | | | | | | |
| Yes | *Reference* | |  |  |  |  |
| No | 1.16 (0.82-1.65) | | 0.394 |  | 1.09 (0.74-1.60) | 0.673 |
| **COVID-19 infection** |  | |  |  |  |  |
| No | *Reference* | |  |  |  |  |
| Yes | 5.89 (1.75-19.77) | | **0.004** |  | 4.38 (1.39-13.76) | **0.012** |
| **Someone close infected e.g. A friend, a family member** | | | | | | |
| No | *Reference* | |  |  |  |  |
| Yes | 1.91 (1.26-2.89) | | **0.002** |  | 1.85 (1.18-2.90) | **0.007** |
| **Parents and peer relationships** |  | |  |  |  |  |
| **Hear from or talk to your friends** |  | |  |  |  |  |
| Rarely/almost never | *Reference* | |  |  |  |  |
| Occasionally | 0.50 (0.34-0.73) | | **<0.001** |  | 0.59 (0.39-0.90) | **0.013** |
| Frequently | 0.45 (0.30-0.68) | | **<0.001** |  | 0.51 (0.32-0.81) | **0.004** |
| **Change of interaction with peers since last week** | | | | | | |
| No – it is the same | *Reference* | |  |  |  |  |
| Yes-I interact with them less | 2.27 (1.59-3.22) | | **<0.001** |  | 2.51 (1.70-3.70) | **<0.001** |
| Yes- I interact with them more | 1.07 (0.66-1.73) | | 0.795 |  | 1.08 (0.62-1.89) | 0.775 |
| **Feeling lonely/lacking company/feeling left out or isolated** | | | | | | |
| Not at all | *Reference* | |  |  |  |  |
| Sometimes | 2.96 (2.07-4.24) | | **<0.001** |  | 2.94 (1.98-4.38) | **<0.001** |
| Always | 17.10 (8.61-33.94) | | **<0.001** |  | 10.48 (4.68-19.33) | **<0.001** |
| **Close with your parents** |  | |  |  |  |  |
| Not very close/distant | *Reference* | |  |  |  |  |
| Fairly close | 0.39 (0.22-0.67) | | **0.001** |  | 0.69 (0.39-1.24) | 0.214 |
| Very/extremely close | 0.29 (0.18-0.44) | | **<0.001** |  | 0.41 (0.25-0.66) | **0.001** |
| **Argue with parent** |  | |  |  |  |  |
| Rarely/almost never | *Reference* | |  |  |  |  |
| Occasionally | 1.13 (0.76-1.68) | | 0.532 |  | 1.22 (0.78-1.89) | 0.379 |
| Frequently | 2.17 (1.43-3.27) | | **<0.001** |  | 2.18 (1.39-3.41) | **0.001** |
| **Health risk behaviours** |  | |  |  |  |  |
| **Feeling unsafe**  No | *Reference* | |  |  |  |  |
| Yes | 2.78 (1.98-3.90) | | **<0.001** |  | 2.65 (1.83-3.83) | **<0.001** |
| **Physically forced to have sex**  No | *Reference* | |  |  |  |  |
| Yes | 3.26 (1.87-5.68) | | **<0.001** |  | 3.42 (1.93-6.03) | **<0.001** |
| **Drunk alcohol (at least a bottle within a month ago)** | | | | | | |
| No | *Reference* | |  |  |  |  |
| Yes | 1.89 (1.18-3.03) | | **0.008** |  | 2.09 (1.27-3.45) | **0.004** |
