# Supplementary material for: Mental health and psychological well-being of Kenyan adolescents from Nairobi and the Coast regions in the context of COVID-19
Source: Child Adolesc Psychiatry Ment Health. 2023 May 19;17:63. doi: 10.1186/s13034-023-00613-y (PMC10198601; doi:10.1186/s13034-023-00613-y)
Supplement: Supplementary file 2 — Additional file 2: Table S2. Results from univariate linear model showing the relationship between predictor variables and pandemic anxiety, quality of life, and emotional and behavioural problems. [file 13034_2023_613_MOESM2_ESM.docx]

**Additional table S2:** Results from univariate linear model showing the relationship between predictor variables and pandemic anxiety, quality of life, and emotional and behavioural problems

|  | **Quality of life** | | **Pandemic anxiety** | | **Emotional and behavioural problems** | |
| --- | --- | --- | --- | --- | --- | --- |
|  | ***ß* (Std.Err)** | ***P*-value** | ***ß* (Std.Err)** | ***P*-value** | ***ß* (Std.Err)** | ***P-*value** |
| **Socio-demographic** |  |  |  |  |  |  |
| **Sex** |  |  |  |  |  |  |
| Male | *Reference* |  |  |  |  |  |
| Female | -0.34 (0.45) | 0.447 | 1.12 (0.46) | **0.015** | 1.19 (0.44) | **0.007** |
| **Age** | -0.57 (0.12) | **<0.001** | 0.30 (0.12) | **0.012** | 0.38 (0.12) | **0.001** |
| **Schooling** |  |  |  |  |  |  |
| Yes | *Reference* |  |  |  |  |  |
| No | -1.16 (0.46) | **0.013** | 0.27 (0.47) | 0.572 | 2.54 (0.45) | **<0.001** |
| **Level of education** |  |  |  |  |  |  |
| None | *Reference* |  |  |  |  |  |
| Primary | -1.74 (2.38) | 0.466 | 0.11 (2.42) | 0.965 | -1.17 (2.34) | 0.619 |
| Secondary | -3.14 (2.38) | 0.186 | 0.86 (2.42) | 0.722 | -0.95 (2.34) | 0.684 |
| **Religion** |  |  |  |  |  |  |
| Christian | *Reference* |  |  |  |  |  |
| Islam | 2.70 (0.51) | **<0.001** | -0.77 (0.53) | 0.142 | -1.34 (0.51) | **0.008** |
| Others e.g. traditional | 1.54 (2.77) | 0.579 | 0.53 (2.85) | 0.851 | -1.58 (2.75) | 0.566 |
| **Social Economic Status** | 0.36 (0.14) | **0.012** | -0.14 (0.14) | 0.329 | -0.20 (0.14) | 0.155 |
| **Have any child(ren)** |  |  |  |  |  |  |
| No | *Reference* |  |  |  |  |  |
| Yes | -2.25 (1.05) | **0.033** | 2.27 (1.07) | **0.034** | 0.94 (1.03) | 0.366 |
| **COVID-19 related questions** |  |  |  |  |  |  |
| **Receive support before lockdown (Mental health, support from social services, educational support)** | | | | | | |
| Yes | *Reference* |  |  |  |  |  |
| No | 0.64 (0.48) | 0.184 | 0.15 (0.49) | 0.755 | -0.86 (0.47) | 0.069 |
| **COVID-19 infection** |  |  |  |  |  |  |
| No | *Reference* |  |  |  |  |  |
| Yes | -3.50 (1.82) | 0.055 | 0.59 (1.85) | 0.748 | 5.68 (1.78) | **0.001** |
| **Someone close infected e.g. a friend, a family member** | | | | | | |
| No | *Reference* |  |  |  |  |  |
| Yes | -1.23 (0.63) | 0.051 | 1.32 (0.63) | 0.038 | 2.30 (0.61) | **<0.001** |
| **Parents and peer relationships** |  |  |  |  |  |  |
| **Hear from or talk to your friends** |  |  |  |  |  |  |
| Rarely/almost never | *Reference* |  |  |  |  |  |
| Occasionally | 0.74 (0.53) | 0.167 | -0.32 (0.55) | 0.557 | -1.65 (0.53) | **0.002** |
| Frequently | 3.45 (0.56) | **<0.001** | -0.30 (0.758) | **0.027** | -2.09 (0.56) | **<0.001** |
| **Change of interaction with peers since last week** | | | | | | |
| No – it is the same | *Reference* |  |  |  |  |  |
| Yes-I interact with them less | -2.86 (0.50) | **<0.001** | 1.28 (0.52) | **0.014** | 2.87 (0.49) | **<0.001** |
| Yes- I interact with them more | 0.21 (0.63) | 0.741 | 0.78 (0.65) | 0.231 | 1.00 (0.62) | 0.103 |
| **Feeling lonely/lacking company/feeling left out or isolated** | | | | | | |
| Not at all | *Reference* |  |  |  |  |  |
| Sometimes | -4.26 (0.47) | **<0.001** | 2.51 (0.49) | **<0.001** | 4.97 (0.43) | **<0.001** |
| Always | -4.23 (0.86) | **<0.001** | 4.45 (0.90) | **<0.001** | 9.18 (0.79) | **<0.001** |
| **Close with your parents** |  |  |  |  |  |  |
| Not very close | *Reference* |  |  |  |  |  |
| Fairly close | 0.85 (0.82) | 0.297 | 0.08 (0.84) | 0.925 | -2.67 (0.78) | **0.001** |
| Very/extremely close | 2.91 (0.66) | **0.001** | -1.15 (0.68) | 0.093 | -4.86 (0.64) | **<0.001** |
| **Argue with parent** |  |  |  |  |  |  |
| Rarely/almost never | *Reference* |  |  |  |  |  |
| Occasionally | -1.94 (0.54) | **<0.001** | -0.18 (0.55) | 0.745 | 1.57 (0.52) | **0.003** |
| Frequently | -2.71 (0.61) | **<0.001** | 0.43 (0.63) | 0.495 | 3.90 (0.59) | **<0.001** |
| **Psychosocial stressors** |  |  |  |  |  |  |
| **Feeling unsafe** |  |  |  |  |  |  |
| No | *Reference* |  |  |  |  |  |
| Yes | -2.58 (0.49) | **<0.001** | 1.36 (0.50) | **0.007** | 4.42 (0.46) | **<0.001** |
| **Physically forced to have sex** |  |  |  |  |  |  |
| No | *Reference* |  |  |  |  |  |
| Yes | -2.56 (0.87) | **0.003** | 1.85 (0.89) | **0.037** | 4.66 (0.84) | **<0.001** |
| **Drunk alcohol (at least a bottle within a month ago)** | | | | | | |
| No | *Reference* |  |  |  |  |  |
| Yes | -2.44 (0.72) | **0.001** | 0.29 (0.73) | 0.688 | 2.50 (0.70) | **<0.001** |
